# *Stachys sieboldii* Extract Supplementation Attenuates Memory Deficits by Modulating BDNF-CREB and Its Downstream Molecules, in Animal Models of Memory Impairment

**DOI:** 10.3390/nu10070917

**Published:** 2018-07-17

**Authors:** Vijaya Abinaya Ravichandran, Mina Kim, Seong Kyu Han, Youn Soo Cha

**Affiliations:** 1 Department of Food Science and Human Nutrition, Chonbuk National University, 664-14 Duckjin-dong, Jeonju, Jeonbuk 561-756, Korea; vijayaabinaya07@gmail.com; 2 Division of Functional Food and Nutrition, Department of Agrofood Resources, National Institute of Agricultural Science, Rural Development Administration, Wanju 55365, Korea; lucidminakim@gmail.com; 3 Department of Oral Physiology, School of Dentistry and Institute of Oral Bioscience, Chonbuk National University, Jeonju 561-756, Korea; skhan@jbnu.ac.kr

**Keywords:** *Stachys sieboldii*, dementia, memory loss, cholinergic neurotransmission, neuroplasticity targets, GABA_A_ receptor

## Abstract

Cholinergic dysfunction, impaired brain-derived neurotrophic factor and cAMP response element binding protein (BDNF-CREB) signaling are one of the major pathological hallmarks of cognitive impairment. Therefore, improving cholinergic neurotransmission, and regulating the BDNF-CREB pathway by downregulating apoptosis genes is one strategy for inhibiting the etiology of dementia. This study evaluates the potential effects of *Stachys sieboldii* MIQ (SS) extract against cognitive dysfunction and its underlying mechanisms. SS supplementation for 33 days improved scopolamine-induced memory impairment symptoms in Morris water maze test and Y-maze test. SS reduced the acetylcholineesterase activity and significantly increase acetylcholine and cholineacetyltransferase activity in the brain. In the subsequent mechanism study, SS regulated the mRNA expression level of neuronal plasticity molecules such as (nerve growth factor) NGF, BDNF, CREB, and its downstream molecules such as Bcl-2 and Egr-1 by downregulating the neuronal apoptosis targets in both hippocampus and frontal cortex. Additionally, inward currents caused by SS in hippocampal CA1 neurons was partially blocked by the GABA receptor antagonist picrotoxin (50 μM), suggesting that SS acts on synaptic/extrasynaptic GABA_A_ receptors. These findings indicate that SS may function in a way that is similar to nootropic drugs by inhibiting cholinergic abnormalities, and neuronal apoptosis targets and ultimately increasing the expression of BDNF-CREB.

## 1. Introduction

Dementia is a current epidemic (approx. 46.8 million new cases worldwide in 2015) and public health and economic burden that attracts increasing investment into research [1]. The disease is marked by a slow, progressive, and irreversible decline in neurocognitive functioning over the years that affects memory, language, and problem-solving abilities [2]. Accumulating evidence and post-mortem studies of the brains of patients with dementia revealed low levels of the acetylcholine (ACh) and choline acetyltransferase (CAT) and increased level of acetylcholinesterase (AChE) [3,4]. Therefore, cholinergic abnormalities have received specific attention and most therapies for dementia are directed to this system [5].

Numerous molecules related to cognitive function have been identified recently. Among them, the first discovered neurotrophin, nerve growth factor (NGF), regulates biological mechanisms related to neuronal plasticity in the brain [6]. The second discovered neurotrophin is brain-derived neurotrophic factor (BDNF), and its major receptor tropomyosin receptor kinase B (TrkB) are involved in synaptic plasticity and memory processes in both the hippocampus and cortical neurons [7]. BDNF is also involved in long term memory (LTM) formation in the CA1 region of the hippocampus, via the activation of the transcription factor cAMP response element binding protein (CREB) [8]. CREB leads to neuronal survival through the regulation of downstream target genes such as B-cell lymphoma 2 (*Bcl2*) [9] and mediates neuronal plasticity by early growth response 1 (EGR1) [10]. Conversely, neuronal apoptosis carried out by two main pathways, one of which is the intrinsic pathway driven by Bax leading to mitochondrial outer membrane permeabilization (MOMP) [11]. The increase in MOMP results in release of cyclooxygenase-2 (COX-2) into the cytoplasm [12]. This, in turn, binds to caspase-9 (extrinsic pathway activates), which, recruits and activates the executioner caspase-3 leading to neuronal cell death [13]. These neuronal metabolic pathways demonstrate the possibility that dementia could be ameliorated or prevented by activation of neurotrophic factors or/and by inhibition of neuronal apoptosis.

γ-aminobutyric acid (GABA) begins as the key excitatory neurotransmitter in newly forming circuits, with chloride efflux from GABA type A receptors (GABA_A_Rs) producing membrane depolarization, dendritic outgrowth, and synaptogenesis [14]. GABA_A_R activation also controls the formation and plasticity of GABAergic synapses, results in the normalizing the cognitive impairment [15]. In addition, pharmacological findings show that traditional drugs acting at GABAergic receptors have memory active properties [16,17].

Many synthetic drugs have been introduced for the treatment of dementia and ultimately withdrawn from the market due to severe toxicity and side effects. Therefore, alternative approaches, such as the use of natural products for the development of functional foods with neuroprotective effects, have been of great research interest [18]. The genus Stachys (Lamiaceae) includes about 200–300 species in the world. *Stachys sieboldii* is erect, hairy, perennial, stoloniferous herb, which is indigenous to China; and in Japan and Korea it has been cultivated since ancient times [19]. The extracts of the plant have been used as folk medicine against several infections for many decades. Phytochemical investigation of *Stachys sieboldii* have shown the occurrence of flavonoids, diterpenes, phenyl ethanoid glycosides and saponins [20]. They are also a good source of oligosaccharides, proteins, and water-soluble vitamins (vitamin B complex), which are all major contributors to human nutrition [21]. Several reports have detailed the health benefits of *Stachys sieboldii*, such as its anti-inflammatory activity, ability to lower anoxia, immunosuppressive function, antinephritic activity, antioxidant, antimicrobial activity, they also improve memory and discomfort [20,21,22,23].

In agreement, we have previously reported protective effects of *Stachys sieboldii* MIQ (SS) against H_2_O_2_ induced cytotoxicity in SK-N-SH cells (Human neuroblastoma cell line) and memory amelioration in mice [22]. Notably, an underlying molecular mechanism by which SS exert the neuroprotective effect has not yet been studied. Therefore, the aim of the present investigation was to elucidate the underlying mechanisms by which SS ameliorates learning and memory ability in a scopolamine-induced amnesia animal model with particular emphasis on cholinergic neurotransmission. Moreover, whole cell patch clamp assays were also used to examine the direct membrane effect of the SS as well as its effects on GABA currents in hippocampal CA1 neuron.

## 2. Materials and Methods

### 2.1. Preparation of Stachys Sieboldii Extract

*Stachys sieboldii* was supplied by the Farmer’s morning Wholesaler, (Gochang, Jeonbuk, South Korea) and was washed. Then SS were freeze dried at −40 °C for 48 h, and extracted with 20% ethanol at 40 °C for 4 h. The resultant was filtered, concentrated to 128 brix, and again freeze dried at −40 °C for 48 h. The SS extracts were ground with a 50-μm mesh to yield a powdered sample.UHPLC-MS/MS analysis for crude extract of *Stachys Sieboldii* have been added in supplementary materials (Table S1 and Figure S1).

### 2.2. UHPLC-MS/MS

**Figure 1:**
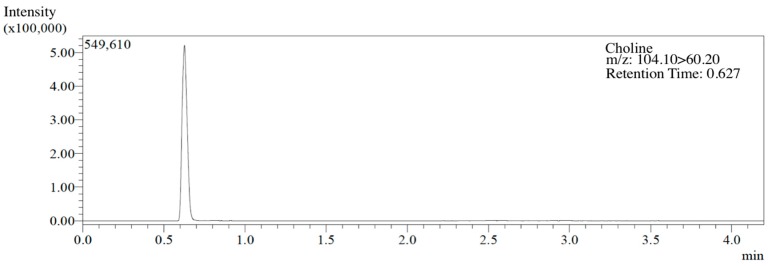
UPLC/MS/MS chromatogram of a *Stachys sieboldii* MIQ extract containing choline.

UHPLC-MS/MS analyses were performed using a Shimadzu UHPLC (SIL-30A) coupled with a triple quadrupole mass spectrometer Shimadzu ESI-MS (8040). Chromatography was carried out using a Waters ACQUITY UPLC^®^BEH HILIC 1.7 μm (2.1 × 100 mm column) at flow rate of 0.5 mL min^−1^, with an injection volume of 1 μL. The mobile phase was formed by solvent A (5 mM ammonium formate, 0.2% formic acid in 50% ACN (acetonitrile)) and solvent B 30 mM ammonium formate, 0.2% formic acid in 50% ACN (acetonitrile). The column temperature was set at 40 °C. The following gradient was used: 0 min, 100% A; 2 min, 100% A; 25 min, 40% B; 40 min, 100% A; 45 min, 100% A. Electrospray ionization (ESI) source, operated in the positive mode and quantitated using selected reaction monitoring (SRM) transitions of m/z 104.1–60.2, 45.3 (Figure 1).

### 2.3. Animals and Experimental Groups

**Figure 2:**
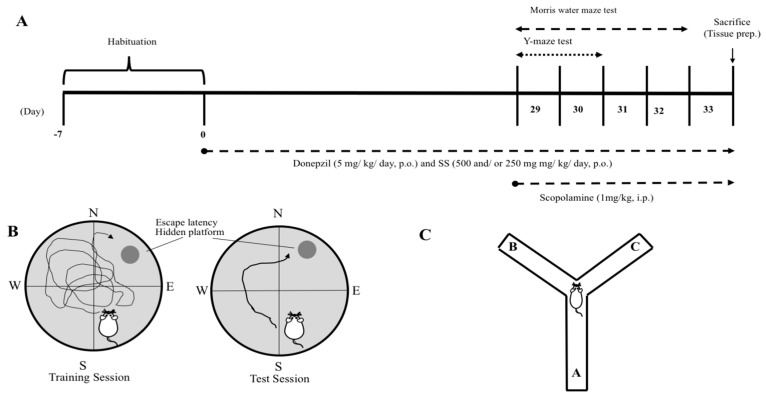
Animal experimental procedure. Schematic representation of the animal experimental protocol (**A**). Memory assessment paradigms used in this study Morris water maze (**B**) and Y-maze (**C**).

All animal procedures were approved by the Animal Care and Use Committee of Chonbuk National University (CBNU 2016–42). Male Sprague–Dawley rats and ICR mice were purchased at 5 weeks of age (Charles River Laboratory, Tokyo, Japan). The animals were housed in individual cages with free access to water and commercial AIN-76A diet (Research Diets, New Brunswick, NJ, USA) in a room with a 12 h/12 h light-dark cycle, at temperature of 23 ± 1 °C, and humidity of 50 ± 5%. After a one-week adaptation period, animals were randomly divided into four groups (*n* = 10 per group): (1) control group (C) (vehicle intraperitoneal (i.p.) + vehicle per os (p.o.)); (2) Scopolamine group (Scop) (scopolamine i.p. + vehicle p.o.) (Sigma Aldrich, St. Louis, MO, USA); (3) Donepezil group (D + Scop) (positive control) [24] (Abcam, Cambridge, UK) (scopolamine i.p. + Donepezil 5 mg/kg body weight p.o.); and (4) SS group (SS + Scop) (scopolamine i.p. + SS 500 and/or 250 mg/kg body weight p.o.). Morris water maze study was conducted in rats with SS concentration of 250 mg/kg body weight p.o. which is equal to 500 mg/kg body weight p.o. in mice according to the equivalent surface area dosage conversion factors [25]. Both donepezil and the SS extract were dissolved in distilled water immediately before use and orally administered to animals for continuous 28 days (pretreatment) at a dose of 5250, or 500 mg/kg body weight, respectively for both Morris water maze and Y-maze experiments. Memory impairment was induced by scopolamine (SCO) treatment (1 mg/kg body weight, i.p.) 30 min before each task. In the control group, the vehicle solution (saline, i.p.) was administered using the same time schedule (Figure 2A). The 200 mg of SS extract contains 0.0676 mg/g choline concentration of dry weight.

### 2.4. Morris Water Maze Test

The Morris water maze task was performed based on the paradigm of Morris, 1985 with slight modifications. (Figure 2B) [26]. The experimental apparatus consisted of a circular pool (diameter, 100 cm; height, 135 cm) containing water at 22 ± 1 °C that was rendered opaque by the addition of powdered milk. The pool was equally divided into quadrants. A platform was positioned inside the tank with its top submerged 1 cm below the water surface in the target quadrant of the maze. In our experiment we have assessed the acquisition in terms of latency to locate the escape platform. The test is based on two phases; the acquisition phase (Training days) and the retention phase (Probe trial). Initially, the training session was performed during which each rat was placed into the water facing toward the wall of the tank. After placing, 300 s were given to each animal to find and mount onto the hidden platform. If it failed to locate the platform during the allocated time, then it was guided gently to the platform and allowed to stay on it for 30 s. Each rat received one training sessions and was repeated 2 times at 10 min intervals for three consecutive days in acquisition training. The probe trial was performed on 4th day after the training phase. The video was recorded using the video tracking system (Ethovision System, Noldus, Wageningen, The Netherlands). Thirty minutes before experiment rats were intraperitoneally injected with scopolamine (1 mg/kg, i.p.).

### 2.5. Y-maze Test

The Y-maze is a three-arm horizontal maze with an angle of 120 degrees, which were 28 cm length, 6 cm width, and 18 cm height. The maze floor and walls were constructed with white polyvinyl plastic. Mice were initially placed in one arm, and then the sequence and number of arm entries were monitored for an 8-min period. An actual alternation was defined when a mouse entered into all three arms on consecutive choices (i.e., ABC, BCA, or CAB, but not CAC, BAB, or ABA). The spontaneous alternation (%) was derived from the total number of alternations divided by the total number of arm entries minus two, which was multiplied by 100 as shown in the following equation: % Alternation = [(Number of alternations)/(Total number of arm entries − 2)] × 100. The number of arm entries also served as an indicator for movement and locomotor activity (Figure 2C) [27].

### 2.6. Sample Collection

At the end of the behavioral test, animals were decapitated immediately, to collect brains. All brain samples were careful excised, rinsed, and immediately stored at −70 °C until analysis was done.

### 2.7. Determination of Acetylcholine, Choline Acetyl Transferase, and Acetyl Choline Esterase Levels in Animal Model of Impaired Memory

ACh, AChE, and CAT levels were analyzed in the hippocampus and cortex of the collected brain sample. The hippocampus and cortex were separated, rinsed in cold PBS, resuspended in PBS, and homogenized. The homogenates were centrifuged at 1500× *g* for 15 min at 4 °C. The supernatants were used in an ACh, AChE, and CAT assay using commercially available kits (MyBioSource, San Diego, CA, USA).

### 2.8. Quantitative Reverse Transcription PCR

Total RNA was isolated from hippocampus and cortex using Trizol reagent (Life Technologies, Inc., Carlsbad, CA, USA). The concentration and purity of RNA was measured spectrophotometrically on a Biodrop Duo instrument (Biochrom, Holliston, MA, USA) calculating the 260:280 absorbance ratio. The extracted RNA was reverse transcribed into cDNA using a high capacity cDNA reverse transcription kit (Applied Biosystems, Foster City, CA, USA). RNA expression levels were quantified using SYBR Green real-time PCR master mix (TOYOBO, Osaka, Japan). Thermocycling was performed on a 7500 Real Time PCR system (Applied Biosystems). Primers used are given in Table 1.

### 2.9. Brain Slice Preparation and Electrophysiology

Brain slices were prepared as described by [17] earlier study with slight modifications in position of brain used. Immature ICR male mice (5–20 Postnatal days) were sacrificed by cervical dislocation between 10:00 and 12:00 h and their brains rapidly removed and placed in the ice-cold low calcium (0.5 mM), high magnesium (6 mM) bicarbonate-buffered artificial cerebrospinal fluid (ACSF) (pH 7.4 when bubbled with 95% O_2_ and 5% CO_2_)). Brains were then cut into 150 to 200 μm-thick coronal slices using a vibratome (Microm, Walldorf, Germany). Patch pipettes (4–6 MΩ) were pulled from thin-wall borosilicate glass-capillary tubing (outer diameter, 1.5 mm; inner diameter, 1.17 mm) (PG52151-4, WPI, Sarasota, FL USA) on a horizontal Flaming/Brown puller (P-97; Sutter Instruments Co., Novato, CA, USA). The pipette solution was passed through a disposable 0.22 μm filter [28]. The whole cell patch clamp recordings were performed under voltage clamp using an Axopatch 200B (Axon Instruments, Union City, CA, USA). The cells were voltage clamped at-60 mV after nullifying the junction potential between the patch pipette and bath solution. The changes in membrane current were sampled online using a Digidata 1322A interface (Axon Instruments, Union City, CA, USA). Acquisition and subsequent analysis of the acquired data were performed using Clampex 9 software (Axon Instruments, USA). All recordings were made at room temperature.

### 2.10. Statistical Analysis

Behavioral studies were analyzed by one-way ANOVA using SPSS version 17.0 (SPSS Institute, Chicago, IL, USA). Values are expressed as means ± standard deviations. The significance of differences between groups was assessed using Tukey’s test. Electrophysiological studies values are expressed as the mean ± S.E.M. One-way ANOVA was performed to analyze more than two experimental groups using origin 7.0. Student’s *t*-test was used to examine the differences between the two experimental groups. *p* < 0.05 was considered significant.

## 3. Results

### 3.1. Effects of SS on Memory Function in Animal Model of Impaired Memory

**Figure 3:**
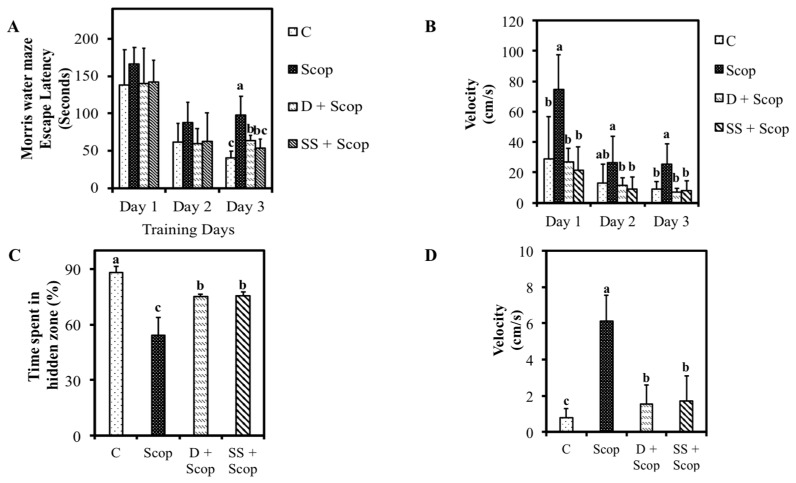
Effect of *Stachys sieboldii* in scopolamine induced memory impairment was assessed by Morris water maze. SS reduced escape latency (**A**), increased velocity (**B**), time spent in hidden zone during probe trial (**C**) and velocity (**D**) All values are mean ± SD (*n* = 10). Mean with different superscripts in the same row indicates significant difference by ANOVA with Tukey’s test at *p* < 0.05 compared to control group.

**Figure 4:**
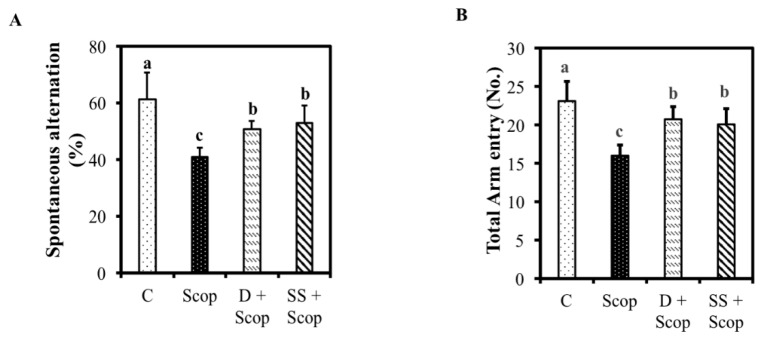
Effect of *Stachys sieboldii* in scopolamine induced memory impairment was assessed by Y-maze. SS reduced spontaneous alternation (%) (**A**), and Total arm entry (No.) (**B**). All values are mean ±SD (*n* = 10). Mean with different superscripts in the same row indicates significant difference by ANOVA with Tukey’s test at *p* < 0.05 compared to control group.

The dose of choline that was administered to the animals was 0.169 mg/day [25]. The effects of SS administration following scopolamine induced memory impairments in animals are shown in Figure 3 and Figure 4. The Control group showed the ability to learn normally, as seen by a significantly decreased in escape latency of day 3 compared to day 1 and day 2 compared to scopolamine treated group (*p* < 0.05) (Figure 3A). However, pretreatment with SS prevented the impairment effects of scopolamine on memory as evidenced by a significant decrease in escape latency time (day 3) in the SS + Scop group compared to the scopolamine alone treated group (*p* < 0.05). We further investigated swimming speed (i.e., velocity) by dividing the escape path length by the escape latency period (Figure 3B). A significant increase in velocity was observed in scopolamine group compared to other groups in all three days. The probe trial was performed 24 h after the last training session (Figure 3C,D). Compared to scopolamine treated group, animals treated with SS and donepezil remained longer time in the target quadrant (*p* < 0.05). The SS + Scop group also showed a significantly decreased velocity (two-fold) compared to the animals treated with scopolamine alone. These results indicate that SS improved spatial memory.

In a Y-maze test, a higher alternation rate demonstrates more sustained cognition, as the animals do not reenter the same arm. Scopolamine group exhibited a significantly decreased spontaneous alternation percentage (40.74% ± 3.52%) compared with the control group (61.6% ± 9%) (*p* < 0.05). Nevertheless, we observed a remarkable increase in working memory in the SS + Scop treated group (*p* < 0.05), indicated by an increase in spontaneous alternation percentage, compared to the scopolamine-treated group (Figure 4A). Additionally, the number of arm entries was recorded and used as an indicator of locomotor activity. As shown in Figure 4B, scopolamine group had a significantly lower number of arm entries compared to the control group; however, animals treated with donepezil and the SS showed increased number of arm entries compare to scopolamine treated group (*p <* 0.05).

### 3.2. Effects of SS on Hippocampal and Frontal Cortical, ACh, CAT, and AChE Levels in Animal Model of Impaired Memory

**Figure 5:**
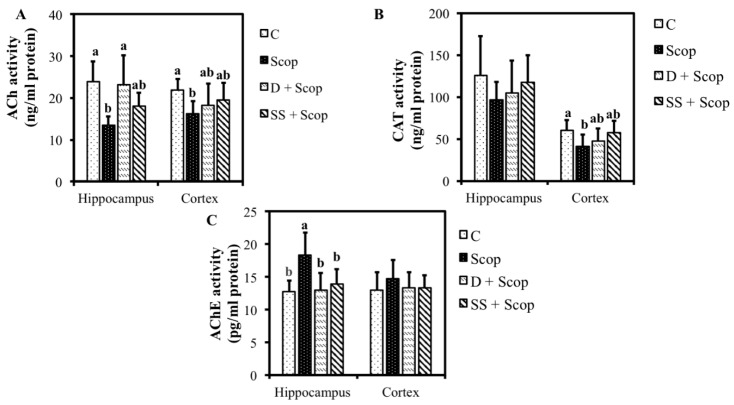
Effect of *Stachys sieboldii* in hippocampus and frontal cortex of animal brain Acetylcholine (**A**) Choline acetyl transferase, (**B**) and Acetylcholine esterase level (**C**). All values are mean ± SD (*n* = 10). Mean with different superscripts in the same row such as a, b indicates significant difference by ANOVA with Tukey’s test at *p* < 0.05 compared to control group.

The levels of ACh, CAT, and AChE in the hippocampus and frontal cortex are presented in Figure 5. Compared with the control group, the scopolamine group exhibited decreased level of ACh in both the hippocampus and cortex (*p* < 0.05). Treatment with either donepezil or SS resulted in a significantly increased level of ACh in animals compared to animals treated with scopolamine alone (Figure 5A). As shown in Figure 5B, CAT levels in the hippocampus did not differ significantly among the groups; however, CAT level showed increased tendency in the donepezil- and SS-treated animals compared to the scopolamine group. Cortical levels of CAT were increased significantly in the control group than in the scopolamine group. Furthermore, in animals treated with either donepezil or SS significantly increased the CAT level in the cortical area compared to scopolamine group (*p* < 0.05). The scopolamine-treated group showed an increased level of AChE compared to the control group in both the hippocampus (*p* < 0.05) and cortex (*p* > 0.05). Interestingly, animals treated with either donepezil or SS showed markedly similar levels of AChE to the control group in both the hippocampus (*p <* 0.05) and cortex (*p* > 0.05) (Figure 5C).

### 3.3. Effect of SS on mRNA Expression in the Hippocampal and Frontal Cortical Area on Memory Impaired Animals

**Figure 6:**
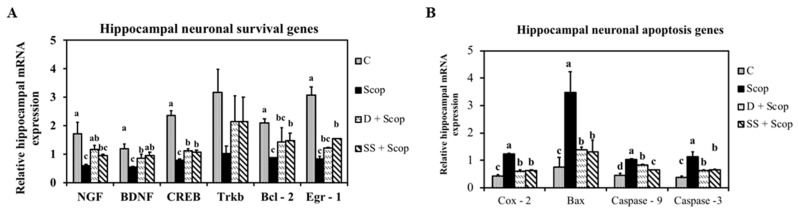
Effect of *Stachys sieboldii* in the mRNA expression of hippocampal neuronal survival genes (**A**) and neuronal apoptosis genes (**B**) All values are mean ± SD (*n* = 4). Mean with different superscripts in the same row such as a, b indicates significant difference by ANOVA with Tukey’s test at *p* < 0.05 compared to control group.

**Figure 7:**
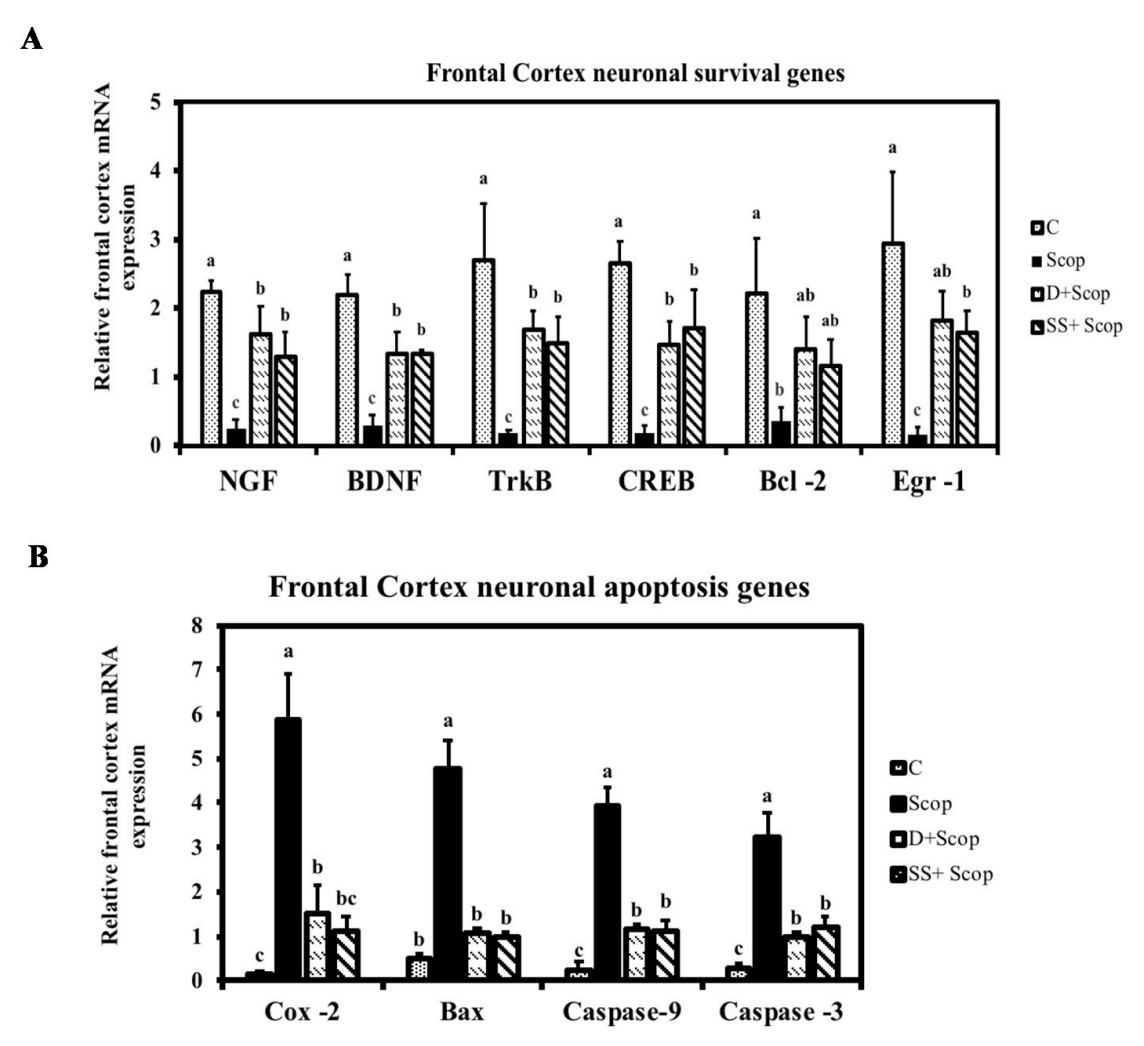
Effect of *Stachys sieboldii* in the mRNA expression of frontal cortical neuronal survival genes (**A**) and neuronal apoptosis genes (**B**) All values are mean ± SD (*n* = 4). Mean with different superscripts in the same row such as a, b indicates significant difference by ANOVA with Tukey’s test at *p* < 0.05 compared to control group.

To understand the mechanisms through which SS protects scopolamine-induced memory impairment, the status of several components of the pathway that regulates neuroplasticity in the hippocampus and frontal cortex was examined using mRNA expression. Scopolamine markedly downregulated NGF, BDNF, and Trkb mRNA expression levels in the hippocampus (Figure 6) and frontal cortex (Figure 7). Meanwhile, SS significantly upregulated a scopolamine-induced decrease in NGF, BDNF, Trkb mRNA expression levels. Furthermore, the expression levels of CREB, Egr-1, and Bcl-2 were investigated in the hippocampus and frontal cortex, as these molecules are implicated in learning and memory processes. Treatment with SS restored the downregulation of CREB, Egr-1, and Bcl-2 induced by scopolamine in the hippocampus and frontal cortex. Simultaneously, Bax, COX-2, caspase-3, and caspase-9 are involved in the neuronal apoptosis pathway in the hippocampus (Figure 6 G–H) and frontal cortex (Figure 7G–H). The mRNA expression levels of Bax, COX-2, caspase-3, and caspase-9 were found to be downregulated in the SS + Scop group compared to the Scopolamine only group. These data demonstrate that the SS attenuates memory-impairment effect induced by scopolamine through activation of NGF-BDNF-CREB signaling in the frontal cortex and hippocampus.

### 3.4. Electrophysiology Results

**Figure 8:**
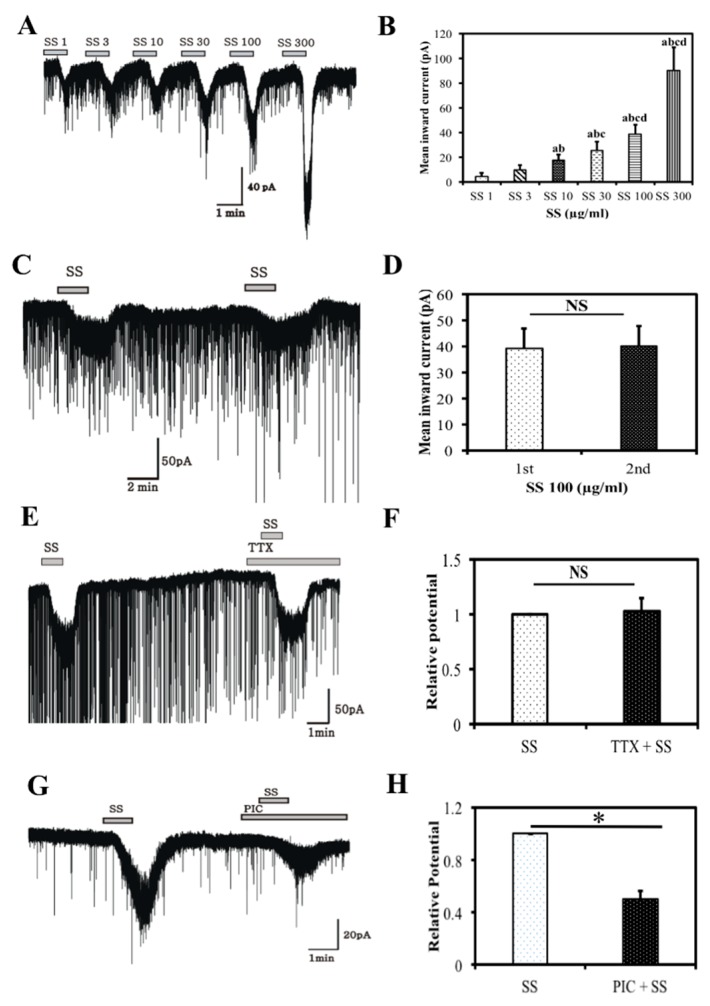
Effect of *Stachys sieboldii* on juvenile CA1 neuron. Concentration-dependent increase in SS-induced inward currents (**A**) and bar graph of Figure A (**B**). Reproducible non-desensitizing effect of SS (100 µg/mL) on a juvenile CA1 neuron (**C**), Bar graph shows the mean relative inward currents induced by SS (100 µg/mL) (**D**). The SS-induced inward currents persisted in the presence of TTX (0.5 μM) (**E**). Bar graph shows the mean inward currents by SS (**F**). The SS-mediated inward currents occurred partly through the extrasynaptic GABA_A_ receptors. A current trace of a juvenile CA1 neuron held at −60 mV, showing presence of slight outward shift in the picrotoxin, (**G**) which got intensified in the presence of SS, (**H**) bar graphs show the mean outward current by PIC alone and PIC in the presence of SS. All values are mean ± S.E.M. (*n* = 5). * Mean with different superscripts in the same row such as a, b, * indicates significant difference among the groups. One-way ANOVA was performed to analyze more than two experimental groups using origin 7.0. Student’s *t*-test was used to examine the differences between the two experimental groups. *p* < 0.05 was considered significant.

Whole cell currents were recorded from CA1 pyramidal neurons in the hippocampal region of immature male mice brains. Under whole-cell, voltage clamp, high chloride pipette solution conditions, SS was applied in concentration-dependent manner. Bath application of 1, 3, 10, 30, 100, or 300 μg/mL of SS revealed a clear, concentration-dependent increase in the SS-induced inward currents (Figure 8A,B) (*p* < 0.05). In another set of experiments, inward currents induced by the application of SS (100 μg/mL) were reproducible, and the response induced by the second application of SS was similar to that of the first application (Figure 8C,D), proving its desensitization effect on CA1 neurons. The effect of SS on CA1 pyramidal neurons was evaluated in the presence of TTX (0.5 μM) to determine if SS acts directly on CA1 pyramidal neurons and not via any action potential-mediated presynaptic release. TTX failed to inhibit the SS-induced inward current in all (5/5) CA1 pyramidal neurons (Figure 8E,F), which shows that SS has a direct effect on CA1 neurons. We also examined whether SS affected the GABA receptors of CA1 pyramidal neurons through successive application of a GABA receptor antagonist. As shown in Figure 8G, the SS-induced inward currents were blocked partially by picrotoxin (PIC), which blocks both synaptic and extrasynaptic GABA_A_ receptors. These results suggest that SS acts on both synaptic and extrasynaptic GABA_A_ receptors (Figure 8H).

## 4. Discussion

The effective concentration, i.e., 500 mg/kg and (or) 250 mg/kg of SS if converted to a human dose is 2430 mg/day containing a choline concentration of 0.8312 mg/day for an average body weight of 60 kg. Two behavioral tests, the Morris water maze and Y-maze tests, are commonly used as experimental tools for examining learning and memory. Scopolamine is a muscarinic antagonist, that affects neurotransmission in the central nervous system and leads to amnesia in animal experimental models [29]. In this study, animals treated with SS + Scop showed a less velocity, decrease escape latency, longer time spent in hidden zones (Figure 3A–D) (spatial memory), and high spontaneous alternation percentage and increase total number of arm entries (working memory) (Figure 4A,B) than the group treated with scopolamine alone [27,29]. The SS improved cognitive function as well as attenuate scopolamine induced memory impairment which was clearly evident in both models of Morris water maze test and Y-maze test. These results suggest that SS could alleviate memory impairment in animal models [30].

Previous studies have shown that basal forebrain nuclei serve as major sources of cholinergic projection neurons to cortex and hippocampus [31,32]. Alteration of cholinergic neurons in the hippocampus and frontal cortex are implicated in the cognitive disorders [3]. Acetylcholine synthesized by choline acetyl transferase in neurons and hydrolyzed by acetylcholine transferase after its release, is essential to memory process [22]. Therapies designed to reverse the cholinergic deficit in large measure based on their importance in cognition. A previous study reported that increased acetylcholine in the hippocampus and frontal cortex during behavioral analysis in an animal model improved memory function following the administration of almond extract [33]. Similar to earlier report, the current study found an increase in acetylcholine content in the hippocampus and frontal cortex in the SS-supplemented groups compared with the scopolamine group. Supplementation with the SS also inhibited AChE (Figure 5C) activity, and increased CAT (Figure 5B) levels in the hippocampus and frontal cortex by modulating the cholinergic system compared to the group treated with only scopolamine. This result again demonstrated that SS protects the brain from memory impairment as observed in the behavioral experiments performed in the present study.

In recent years, investigations into the cellular and molecular pathways related to neuroprotection have been performed, providing a basis for future therapies for memory disorders. NGF plays a critical role in neuronal plasticity [6]. BDNF and its receptor TrkB are implicated in neuronal survival, morphogenesis, and synaptic plasticity [7,34] and also regulates memory formation. CREB leads to the neuronal synaptic plasticity underlying learning and memory through the expression of downstream target genes, such as Bcl2 [9] and EGR1 [10]. Several studies have shown that the expression of BDNF is impaired in patients with dementia, as well as in amnesia induced animal models [8,29,35]. Similar to earlier reports, the current study found that the mRNA expression levels of NGF, BDNF, TrkB, CREB, and other genes such as Bcl2 and EGR1 were downregulated in the scopolamine-treated group compared to the control group. Interestingly, SS supplementation upregulated the expression level of these genes in both the hippocampus (Figure 6A–F) and frontal cortex (Figure 7A–F). Contrarily, neuronal apoptosis genes, such as Bax, lead to MOMP [10] induce the release of COX-2 into the cytoplasm [12], resulting in activation of caspase-9 and caspase-3 and leading to neuronal cell death [11,13]. Studies have reported that an increase in expression of caspases, COX-2, and Bax can promote neuronal degeneration and alleviate the memory loss seen in dementia patients [13,36]. In agreement with these previous studies, upregulation of COX-2, Bax, caspase-9, and caspase-3 was observed in both the hippocampus (Figure 6G–J) and frontal cortex (Figure 7G–J) in the scopolamine group compared to the control group, while downregulation of COX-2, Bax, caspase-9, and caspase-3 was seen in the SS supplemented group. Based on these results, we suggest that SS might prevent memory loss via activation of neurotrophic factors or/and by inhibition of neuronal apoptosis.

The hippocampus is a powerhouse of memory and is essential for converting short-term memory into long-term memory. Specifically, the CA1 region of the hippocampus is involved in this process [28]. In this study, bath application of SS induced reproducible and short lasting inward currents in CA1 neurons (Figure 8C,D). The inward currents persisted in the presence of TTX, a Na^+^ channel blocker, suggesting that SS acts on CA1 neuronal membranes or dendrites directly, rather than by action potential-mediated mechanisms (Figure 8E,F) [28,37]. In addition, the inward currents induced by SS were partially blocked by picrotoxin, a broad GABA_A_ receptor antagonist, suggesting that SS exhibits GABA-mimetic activity via synaptic and extrasynaptic GABA_A_ receptors (Figure 8G,H). This provides evidence that SS acts directly on the GABA binding site of CA1 pyramidal neurons in the hippocampus [17]. These results are in agreement with a previous study [38], which reported that *Withania somnifera* acts at a GABA receptor by inhibiting GABA_A_ receptor agonists. Another study suggested that GABAergic neurons can critically modulate the electrical activity of the hippocampus during the multiple consolidation process of memory storage [39]. This result provides clear evidence that SS has a GABA_A_ receptor activation effect and may play an important role in neuroprotection and neurite outgrowth via regulation and activation of GABA_A_ receptors.

**Figure 9:**
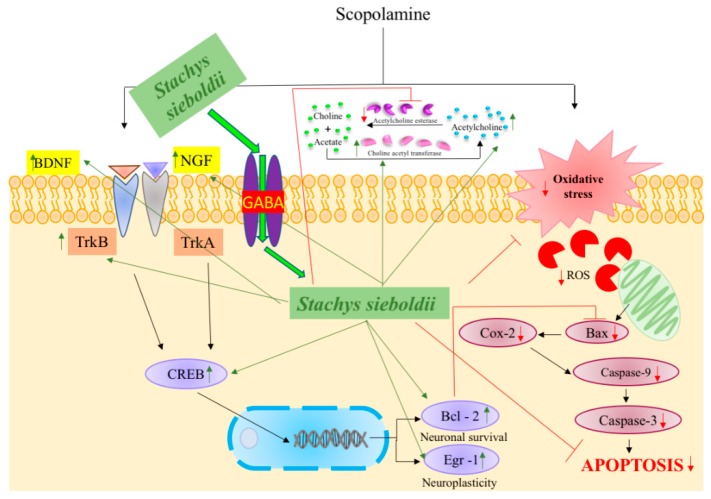
Mechanisms underlying the effects of *Stachys sieboldii* (SS). SS downregulated cellular apoptosis signaling cascade such as Bax, COX-2, Caspase-9 and Caspase-3, which results in the upregulation of memory enhancement-related gene such as NGF, BDNF, TrkB, and CREB, and its downstream targets Bcl-2 and Egr-1 which are involved in neuroplasticity and neuronal survival in the cerebral cortex and hippocampus. SS also reduced the AChE and increased the CAT and ACh in brain. Thus, SS prevents memory impairments through the NGF-CREB-BDNF signaling pathway and inhibitory property of AChE.

In summary, dementia has been reported to be associated with the down regulation of levels of NGF-BDNF-CREB, signaling [30] which in turn it increases the mRNA expression level of apoptotic genes [36] which leads to cholinergic dysfunction and memory deficit [33]. Taken together, the results of this study demonstrated that SS can prevent and protect against scopolamine-induced memory impairment by regulating the NGF-BDNF-CREB signaling pathway (Figure 9) and inhibiting AChE expression. This study also revealed that SS acts through GABA_A_ receptors in CA1 pyramidal neurons of the hippocampus. These data indicate that a daily intake of SS extract via dietary supplementation may produce memory-enhancing effects and prevent dementia in humans. However, further studies are needed to explore the direct mechanism of SS action, along with its clinical safety using consecutive clinical trial.

## Figures and Tables

**Table 1:** Primer sequence used in the animal study.

Gene Name	Primers	Sequence (5′–3′)
NGF	Forward	ACCTCTTCGGACACTCTGG
	Reverse	CGTGGCTGTGGTCTTATCTC
BDNF	Forward	CGAGACCAAGTGTAATCCCA
	Reverse	TCTATCCTTATGAACCGCCA
Trkb	Forward	TCTCATTTTAGGCCGCTTTG
	Reverse	GGGTTTGAGGTGGGTGAAG
CREB	Forward	TACCCAGGGAGGAGCAATAC
	Reverse	GAGGCAGCTTGAACAACAAC
Egr-1	Forward	CCAGTGCCCACCTCTTACTC
	Reverse	TGCAGACTGGAAGGTGCTG
Bcl-2	Forward	TTGACGCTCTCCACACACATG
	Reverse	GGTGGAGGAACTCTTCAGGGA
Bax	Forward	CTGGAAGAAGATGGGCTGAGG
	Reverse	ACCTGAGGTTTATTGGCACCT
Cox-2	Forward	GGCACAAATATGATGTTCGC
	Reverse	CCTCGCTTCTGATCTGTCTTGA
Caspase-3	Forward	AATTCAAGGGACGGGTCATG
	Reverse	GCTTGTGCGCGTACAGTTTC
Caspase-9	Forward	CTGTCCCGTGAAGCAAGGAT
	Reverse	TGGTACATCGGCAGAGAAGC
GAPDH	Forward	TGCACCACCAACTGCTTAGC
	Reverse	GGCATGGACTGTGGTCATGAG

## Supplementary Materials

The following are available online at http://www.mdpi.com/2072-6643/10/7/917/s1, Figure S1: UHPLC-MS/MS of crude extract of Stachys Sieboldii; Table S1: Compounds present in crude extract of Stachys Sieboldii.

## Abbreviations

SS, *Stachys sieboldii*; WHO, World Health Organization; ACh, Acetylcholine; CAT, Cholineacetyl transferase; AChE, Acetylcholineesterase; NGF, Nerve growth factor; BDNF, Brain-derived neurotrophic factor; TrkB, Tropomyosin receptor kinase B; LTM, long term memory; CREB, cAMP response element binding protein; Bcl2, B-cell lymphoma 2; EGR1, early growth response 1; MOMP, Mitochondrial outer membrane permeabilization; COX-2, cyclooxygenase-2 Bax, Bcl2 associated X protein; GABA, γ-aminobutyric acid; TTX, tetrodotoxin; PIC, Picrotoxin.
