# Supplementary material for: Stachys sieboldii Extract Supplementation Attenuates Memory Deficits by Modulating BDNF-CREB and Its Downstream Molecules, in Animal Models of Memory Impairment
Source: Nutrients. 2018 Jul 17;10(7):917. doi: 10.3390/nu10070917 (PMC6073797; doi:10.3390/nu10070917)
Supplement: Supplementary file 1 [file nutrients-10-00917-s001.pdf]

Supplementary File

Materials and method

1. *UHPL/MS-MS of crude extract Stachys sieboldii*

Unbiased metabolomics analysis was performed using an ultra-performance liquid chromatography (UPLC) system (Waters, Milford, USA). The chromatographic separation was carried out using an ACQUITY UPLC HSS T3 column (100 mm × 2.1 mm, 1.8 μm, Waters) with a column temperature of 40 °C and a flow rate of 0.5 ml/min, where the mobile phase contained solvent A (water +0.1% formic acid) and solvent B (acetonitrile +0.1% formic acid). Metabolites were eluted using the following gradient elution conditions: 97% phase A for 0–5 min; 3–100% liner gradient phase B for 5 ~16 min; 100% phase B for 16–17 min; 100–3% reverse liner gradient phase B for 17~19 min; 97% Phase A for 19–25 min. The loading volume of each sample was 5 μl. The metabolites eluted from the column were detected by a high-resolution tandem mass spectrometer SYNAPT G2 Si HDMS QTOF (Waters) in positive and negative ion modes. For positive ion mode, the capillary voltage and the cone voltage were set at 2 kV and 40 V, respectively. For negative ion mode, they were 1 kV and 40 V, respectively. Centroid MS^E mode was used to collect the mass spectrometry data. The primary scan ranged from 50 to 1200 Da and the scanning time was 0.2 s. All the parent ions were fragmented using 20–40 eV. The information of all fragments were collected and the time was 0.2 s. In the data acquisition process, the LE signal was gained every 3 s for real-time quality correction. For accurate mass acquisition, leucine enkephalin at a flow rate of 10 μl min^{-1} was used as a lock mass by a lock spray interface to monitor the positive ($[M + H]^+ = 556.2771$) and the negative ($[M - H]^- = 554.2615$) ion modes. Data acquisition and analysis were controlled by Waters UNIFI V1.71 software. The scan rang in MS and MS/MS modes were over a range of 50–1200 m/z (Figure S1) (Table S1).

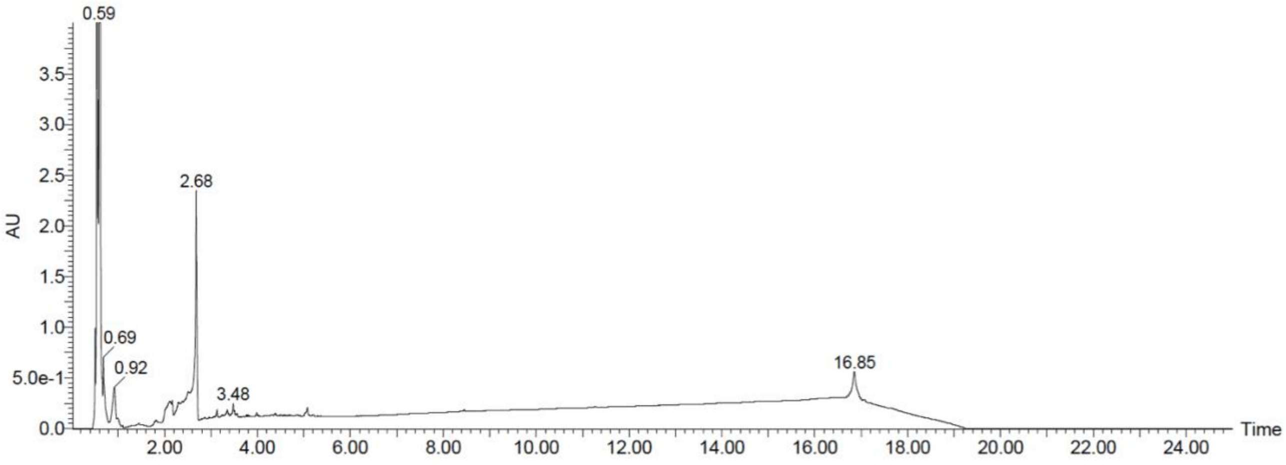

Figure S1: UHPLC - MS/ MS of crude extract of Stachys Sieboldii

Table S1 Compounds present in crude extract of Stachys Sieboldii

Peaks	Compound Name	Retention time (min)
1.	Stachyose	0.59
2.	Rehmannioside A	0.69
3.	Stachydrine	0.92
4.	Umbelliferone	2.68
5.	Coumaric acid	3.48
6.	Proanthocyanidin	16.85
